# Positive intervention effect of mobile health application based on mindfulness and social support theory on postpartum depression symptoms of puerperae

**DOI:** 10.1186/s12905-022-01996-4

**Published:** 2022-10-10

**Authors:** Chao Liu, Hao Chen, Fang Zhou, Qiqi Long, Kan Wu, Liang-Ming Lo, Tai-Ho Hung, Chia-Yih Liu, Wen-Ko Chiou

**Affiliations:** 1 School of Journalism and Communication, Hua Qiao University, School of Film and Communication, Xiamen University of Technology, Department of Economic and Management, Suzhou Vocational Institute of Industrial Technology, Xiamen, 361021 China; 2 Director of Business Analytics Research Center, Chang Gung University, Taoyuan, 33302 Taiwan; 3 Shanghai Obstetrics and Gynecology Hospital, Fudan University, Shanghai, 200090 China; 4 Department of Orthopaedic Surgery, Chang Gung Memorial Hospital, Taoyuan, 33302 Taiwan; 5 Department of Obstetrics and Gynecology, Chang Gung Memorial Hospital, Taipei, College of Medicine, Chang Gung University, Taoyuan City, Taiwan; 6 Department of Psychiatry, Chang Gung Memorial Hospital, Taipei, 10507 Taiwan; 7 Department of Industrial Design, Chang Gung University, Taoyuan, 33302 Taiwan; 8 Department of Industrial Engineering and Management, Ming Chi University of Technology, New Taipei, 24301 Taiwan

**Keywords:** Mobile health application, Mindfulness, Perceived social support, Maternal parental self-efficacy, Postpartum depressive symptoms

## Abstract

**Objective:**

This study investigated the effects of mobile health application designed based on mindfulness and social support theory on parenting self-efficacy and postpartum depression symptoms of puerperae.

**Methods:**

We recruited 130 puerperae from a hospital in China and randomized them to an App use group (n = 65) and a waiting control group (n = 65). The App group underwent an 8-week app use intervention while the control group underwent no intervention. We measured four main variables (mindfulness, perceived social support, maternal parental self-efficacy and postpartum depressive symptoms) before and after the App use intervention.

**Results:**

In the App group, perceived social support, maternal parental self-efficacy were significantly higher and postpartum depressive symptoms was significantly lower. In the control group, there were no significant differences in any of the four variables between the pre-test and post-test.

**Conclusions:**

Our findings indicated that the mobile health application may help to improve perceived social support, maternal self-efficacy and reduce postpartum depressive symptoms. The finding of the mobile health application's effect extends our understanding of integrative effects of mindfulness and perceived social support on reduction of postpartum depressive symptoms and suggests clinical potentials in the treatment of postpartum depressive symptoms.

## Introduction

Postpartum depressive symptoms refer to the obvious depressive symptoms or typical depressive episodes that occur in women during the puerperium. The basic characteristics of postpartum depressive symptoms are sustained emotional distress, accompanied by changes in thinking and behavior and reflected in physical symptoms. It has been reported that postpartum depressive symptoms usually begins 2 weeks after childbirth and peaks in 4–6 weeks [1]. According to the report of the World Health Organization, depression has become one of the major diseases causing disability in the world. Severe postpartum depression may lead to pessimistic suicide and even infanticide [2]. If not treated in time, it will not be conducive to postpartum rehabilitation, increase the economic burden on the family and society. As one of the perinatal complications of postpartum depressive symptoms, reducing postpartum psychological distress and anxiety has become a critical public health goal [3]. According to the previous study, medications are widely used to prevent maternal depression, with more than 7% of women taking antidepressants and at least 75% of women diagnosed with depression recommending antidepressants. However, antidepressants such as paroxetine have been shown to increase the risk of fetal malformation [4, 5], and many mothers are less likely to accept medication for fear of its impact on the safety of their babies and themselves. The study also found that among the puerperae receiving antidepressant treatment, the rate of drug withdrawal was more than 50%, and the recurrence rate of postpartum depression was 70% [6]. Considering the many adverse reactions and side effects of drug therapy, this study aims to develop a safe and convenient non-drug intervention method for postpartum depression.

Some scholars suggest that the long-term and necessary concern for postpartum depression should be expanded to include a more comprehensive assessment of the emotional experience of pregnancy and postpartum [2]. Timely detection of emotion-related problems through specific psychological assessment of negative emotions may help prevent postpartum mental disorders [2]. Rizzo et al. [2] found that agoraphobia, social anxiety, eating problems and other emotion-related mental disorders were positively correlated with the occurrence of postpartum depression and could be used as predictors of postpartum depression. Women may experience depression and anxiety and other negative emotional states during pregnancy, even without mental disorders, these negative emotions may also have a negative impact on postpartum, and the experience and expression of anger can be considered as a susceptibility factor for the onset of postpartum depression [7]. Therefore, early identification and intervention of maternal emotional problems are crucial for the prevention of maternal mental health and postpartum depression.

Based on the strong regulating effect of mindfulness on emotions, the promoting effect of social support and self-efficacy on postpartum depression, this study constructed the research framework according to these theories, and developed mobile phone applications suitable for puerperae to intervene and reduce their postpartum depression.

### Perceived social support and postpartum depressive symptoms

Studies have shown that inadequate perceived social support is a major risk factor for postpartum depressive symptoms [8]. The framework of social support theory is based on social Exchange Theory and self-efficacy theory [9]. Perceived social support is one of the influencing factors that can help predict and reduce the risk of postpartum depressive symptoms. Perceived social support can also indirectly improve parenting self-efficacy and effectively reduce the risk of postpartum depressive symptoms [10]. Social support is broadly defined as the physical, cognitive and emotional support and assistance provided by members or experts in a social network [11]. Social support is also defined as structural support and functional support. Structural support refers to the degree of connection between the supported and its social network, such as increasing the number of people in the social network, the frequency of interaction with people, and participating in social activities; Functional support is divided into four functions: emotional support, tangible support, informational support and appraisal support [12]. Social support can be divided into actual received social support and perceived social support. Perceiving social support emphasizes the consistency between the effect of received social support and the degree of perceived support, and pays attention to self-understanding and self-feeling of social support [13]. Perceived social support found to be a better predictor of maternal mental health than received social support [14, 15].

The lack of perceived social support is the most significant risk factor for postpartum depressive symptoms. Some patients with postpartum depressive symptoms often magnate their pain and adverse factors and fail to see their advantages. Improving perceived social support and the utilization of perceived social support can effectively reduce the occurrence of postpartum depressive symptoms. Research indicates that perceived social support affects postpartum depressive symptoms through self-efficacy, and self-efficacy is the mediating factor between perceived social support and postpartum depressive symptoms. Enhancing perceived social support can help improve self-efficacy and reduce the occurrence of postpartum depressive symptoms [10]. Good perceived social support can help puerperae maintain a positive attitude, reduce their psychological pressure, and significantly reduce the incidence of postpartum depression symptoms. The more perceived social support they have, the more they can make full use of it. For example, if someone helps the mother take care of the child and daily life, and visits and chats regularly, the physical fatigue and mental helplessness of the mother can be reduced, and the incidence of postpartum depressive symptoms is mild or even does not occur [16].

Parenting self-efficacy refers to the self-efficacy in the work of raising infants and young children, and is a judgment or belief made by the puerperae on their own ability in the work of raising infants and young children [17]. Research suggests that childbirth and role adjustment cause moderate to severe stress for most puerperium mothers. Most of the puerperae are primiparas, and childbirth and the birth of a newborn, as a strong stress mode, may cause role transformation problems after childbirth due to stress, fatigue, pain, etc., and then produce more serious psychological problems, such as postpartum depressive symptoms, sleep disorders, etc. [18]. These can not only damage maternal health, but also may lead to maternal failure to correctly understand the behavioral changes of infants and children, and make correct judgments and treatments on these behaviors, leading to the reduction of parenting self-efficacy [19].

### Mindfulness and postpartum depressive symptoms

Some scholars have studied the effect of mindfulness therapy on postpartum depressive symptoms in order to alleviate the symptoms of postpartum depressive symptoms. The concept of mindfulness originally came from Buddhist meditation. Kabat-zin defined mindfulness as a purposeful and conscious focus on the present without judgment on all concepts of the present [20]. The Canadian Network for Mood and Anxiety (CANMAT) guidelines recommend that mindfulness therapy be used as an adjunct treatment for acute major depression, as well as a primary treatment for relapse prevention. At present, more mature mindfulness-based therapies include mindfulness-based stress reduction therapy and mindfulness-based cognitive therapy. The intervention effect of mindfulness therapy on patients with postpartum depressive symptoms is significant, whether the patients who receive mindfulness training to prevent postpartum depressive symptoms before birth, or the patients who receive mindfulness therapy after birth, as the primary treatment or adjuvant method, can effectively reduce their depression symptoms [21]. After mindfulness training, one does not treat life stress as a difficulty or disaster, but rather as an adjustable way to relieve emotional stress. Through 8–12 weeks of mindfulness training, the emotional information of postpartum depressive symptoms patients can be offset, and the negative memories can be occupied by positive emotions such as mindfulness, kindness and compassion, so as to get rid of the normal uncomfortable thinking pattern, and promote the puerperae to adapt to the new life and new role as soon as possible [22]. Mindfulness therapy can reduce the impulsiveness of behavior in a variety of ways to increase awareness of one's own experience and thus enhance control of impulsive behavior, which allows women to maximize their behavior and reduce the anxiety and discomfort associated with role change. Clinical controlled studies have shown that mindfulness therapy is effective in the intervention of postpartum depressive symptoms, MBSR can reduce postpartum depressive symptoms of puerperae [23], MBCT can reduce postpartum depressive symptoms of puerperae and negative emotions of partners [24], and the effect of MBSR combined with depression medication is more significant than that of depression medication alone [25]. Eight weeks MBCT intervention reduced maternal postpartum depressive symptoms, which is first understand the cause of the maternal depression through communication, then targeted them with mindfulness training, by changing their cognition, help them change roles, and through the relaxation training for patients with psychological counseling, to improve the psychological status of patients [26]. Mindfulness cognitive therapy can decrease postpartum depressive symptoms. The results showed that this therapy reduced the maternal depression score from 24.75 to 18.5, and there was no significant difference in the control group, indicating the effectiveness of this therapy [27]. After eight weeks of transcendental meditation intervening women with postpartum depressive symptoms, researchers found that the depression scores of the intervention group were not only lower than those of the control group after the intervention, but also lower than those of the control group at eight weeks of follow-up, and the difference was statistically significant [25]. The researchers used group mindfulness-based cognitive therapy as an intervention to postpartum depressive symptoms, and achieved good results [28].

Compared with other therapies, mindfulness based psychotherapy has well-structured and standardized intervention contents and topics, so that the intervention can be easily performed by a formally trained facilitator, is more operational, and has greater clinical significance for psychological care [24]. Therefore, it is necessary to reduce maternal depression by improving the level of mindfulness of mothers with postpartum depressive symptoms. Despite all the benefits of mindfulness therapies, many mothers do not benefit from them because they require highly qualified teachers, involve multiple face-to-face training sessions, and can impose significant time and financial costs on mothers [20, 29]. The development of mobile health (mHealth) overcomes many obstacles associated with traditional mindfulness meditation training.

### The mHealth and postpartum depressive symptoms

The World Health Organization defines mHealth as medical treatment and health management through mobile devices, such as mobile phones, patient monitoring devices, handheld computers and other wireless devices [30]. The emerging technology of mHealth has attracted the attention of medical staff, psychologists, social workers and other researchers in the field of health promotion. As a supplement to traditional medical methods, mHealth technology obviously has its own advantages. Its two basic characteristics are high convenience and low cost. The convenience of mobile devices eliminates spatial barriers to a certain extent, enabling users to obtain mHealth services from any place with access to the Internet [31]. At the same time, from the perspective of cost efficiency, compared with face-to-face consultation or clinical medical care, mHealth technology shows higher cost efficiency. The feature of breaking through space restrictions can greatly save the time and transportation costs of users and medical professionals [32]. The rapid technological revolution of mHealth and its widespread use, especially in low- and middle-income countries in recent years, make it a promising component of the global delivery of patient-centered care, and its potential has been recognized by the WHO [33].

The mHealth technology services in the postpartum depressive symptoms area range from SMS services and telephone communication to web-based online interventions. With the wide popularity of smart phones, relevant researches are more inclined to the application of mobile applications [34]. The study found that women of childbearing age were more likely to turn to the cellphone for health information, and that new mothers with postpartum depressive symptoms felt they could use mHealth technology to help them manage their symptoms of depression [35]. The researchers turned their attention to the role of mHealth interventions in preventing and treating postpartum depressive symptoms, and the study showed promising results in reducing postpartum depressive symptoms. To date, several studies have assessed the potential and efficacy of telemedicine tools in supporting new mothers with postpartum depressive symptoms. mHealth interventions are effective for both prevention and treatment of postpartum depressive symptoms [36, 37]. MHealth measures to prevent postpartum depressive symptoms include: telephone-based educational conferences, mHealth app, and telephone-based support. MHealth interventions used to treat postpartum depressive symptoms include: technology-based peer support interventions, mobile app, phone-managed CBT, phone-based support, and phone-based physical activity interventions [38]. However, there is currently no mHealth that combines both mindfulness and perceived social support to intervene in postpartum depressive symptoms.

## Research gaps, research objectives and hypotheses

Few studies have explored the effects of apps that combine both mindfulness and perceived social support on maternal parenting self-efficacy and postpartum depressive symptoms, and this study attempted to fill this gap based on systems thinking. Therefore, the aim of this study was to test whether a mHealth application combining mindfulness and perceived social support could improve maternal self-efficacy and reduce postpartum depressive symptoms. Based on mindfulness and social support theory, We develop a mHealth application named ‘‘We'll App’’. There are mindfulness meditation training courses in the We'll App to help puerperae meditate, regulate their emotions and improve their mindfulness. There is also a function for puerperae to make wishes, so that relatives and friends can timely understand the needs of them, and to provide the corresponding social support. It also contains a lot of parenting knowledge to help improve maternal self-efficacy.

Based on the above literature theory and research findings, this study puts forward the following hypotheses:

### Hypothesis 1 (H1):

We’ll App intervention can significantly reduce the level of postpartum depressive symptoms of the subjects.

### Hypothesis 2 (H2):

We’ll App intervention can significantly improve the level of parenting self-efficacy of subjects.

### Hypothesis 3 (H3):

We’ll App intervention can significantly improve the level of perceived social support of subjects.

### Hypothesis 4 (H4):

We’ll App intervention can significantly improve the level of mindfulness of subjects.

## Methods

### Participants

The present study involved 130 pregnant women about to give birth in a hospital in China, the age ranges from 25 to 40 years old. On the premise of matching various demographic characteristics as much as possible, the subjects were randomly assigned to two groups: the App group (65participants) and the waiting control group (65 participants). The demographic characteristics of the two groups are shown in Table 1, and Chi-square test results showed that no significant differences in these demographic characteristics between the two groups.

**Table 1:** Demographic characteristics of participants

	Total (n = 130)	App group (n = 65)	Control group (n = 65)	χ^2^	*p*
Characteristic n(%)					
Age (SD)	31.81 (5.36)	30.32 (5.21)	33.29 (5.15)	0.869	0.351
Non-senior age (< 35)	87 (66.9%)	46 (70.8%)	41 (63.1%)		
Senior age (≥ 35)	43 (33.1%)	19 (29.2%)	24 (36.9%)		
Education level					
High school or below	5 (3.8%)	3 (4.6%)	2 (3.1%)	0.647	0.724
University	92 (70.8%)	44 (67.7%)	48 (73.8%)		
Master degree or above	33 (25.4%)	18 (27.7%)	15 (23.1%)		
Employment situation					
Full time	98 (75.4%)	52 (80%)	46 (70.8%)	1.534	0.464
Part time	8 (6.1%)	3 (4.6%)	5 (7.7%)		
Housewife	24 (18.5%)	10 (15.4%)	14 (21.5%)		
Monthly household income					
Under 3000 RMB	23 (16.9)	18 (27.7%)	15 (23.1%)	0.789	0.852
3000–5000 RMB	32 (25.4%)	17 (26.1%)	15 (23.1%)		
5000–10,000 RMB	32 (24.6%)	10 (15.4%)	12 (18.4%)		
Over 10,000 RMB	43 (33.1%)	20 (30.8%)	23 (35.4%)		
Mode of delivery					
Natural birth	89 (68.5%)	48 (73.8%)	41 (63.1%)	1.746	0.186
Caesarean birth	41 (31.5%)	17 (26.2%)	24 (36.9%)		
First production					
Yes	78 (60%)	38 (58.5%)	40 (61.5%)	0.128	0.720
No	52 (40%)	27 (41.5%)	25 (38.5%)		
Planned pregnancy					
Yes	97 (74.6%)	46 (70.8%)	51 (78.5%)	1.015	0.314
No	33 (25.4%)	19 (29.2%)	14 (21.5%)		
Full-term pregnancy					
Yes	116 (89.2%)	60 (92.3%)	56 (86.2%)	1.281	0.258
No	14 (10.8%)	5 (7.7%)	9 (13.8%)		
Postpartum complications					
Yes	18 (13.8%)	7 (10.8%)	11 (16.9%)	1.032	0.310
No	112 (86.2%)	58 (89.2%)	54 (83.1%)		
Baby sex					
Male	72 (55.4%)	34 (52.3%)	38 (58.5%)	0.498	0.480
Female	58 (44.6%)	31 (47.7%)	27 (41.5%)		
Is the baby abnormal					
Yes	12 (9.2%)	8 (12.3%)	4 (6.2%)	1.469	0.226
No	118 (90.8%)	57 (87.7%)	61 (93.8%)		
Feeding mode					
Breast milk	67 (51.5%)	31 (47.7%)	36 (55.4%)	1.065	0.587
Artificial	24 (18.5%)	14 (21.5%)	10 (15.4%)		
Blend	39 (30%)	20 (30.8%)	19 (29.2%)		
Postpartum supplementary parenting					
Without assistance	10 (7.7%)	6 (9.2%)	4 (6.1%)	3.316	0.506
Parents and relatives	36 (27.7%)	14 (21.5%)	21 (32.3%)		
Hire a nanny	33 (25.4%)	18 (27.7%)	15 (23.1%)		
Postpartum care center	17 (13.1%)	7 (10.8%)	10 (15.4%)		
Two or more kinds of auxiliary	34 (26.1%)	20 (30.8%)	15 (23.1%)		

### Instruments

The Mindful Awareness Scale (MAAS). This scale was developed by Brown and Ryan [39] to measure personal traits of mindfulness. The Chinese version of MAAS was adopted by Deng et al. [40], which had the same 15-item single-factor structure using a 6-point Likert scale, with 1 to 6 representing "almost always" to "almost never". A higher score means a higher mindfulness. MAAS is the most widely used tool for measuring mindfulness traits [41]. A large number of studies have shown that MAAS has good reliability and validity in different cultural populations [42]. In the current study, Cronbach's alpha was 0.91.

Multidimensional perceived social support scale (MSPSS). This scale was developed by Zimet to measure perceived social support [43], Hsu et al. translated it into Chinese [44], focus on understanding the individual self-perception of the degree of perceived social support. 7-point Likert scale was used to score the scale. A higher score indicates a stronger awareness of perceived social support (from "strongly disagree" to "strongly agree"). There are 12 items in the scale, the total score is the sum of the scores of each item. The total score range is 12 to 84, a higher score indicates more perceived social support. In this study, Cronbach’s alpha was 0.92.

Perceived maternal parental self-efficacy tool (PMPS). This scale was developed by Barnes and Adamson-Macedo to measure maternal parental self-efficacy [45]. This scale has 20 items in total, including care-taking procedures, evoking behavior, reading behavior and signaling, situational beliefs. It's a self-report scale, in which 4-point Likert scale was adopted, from 1 to 4, representing "strongly disagree" to "strongly agree", and the higher the score, the higher the effectiveness of the mother's parenting. The Chinese version of PMPS was translated by Chen et al. [46]. In this study, Cronbach’s alpha was 0.93.

Edinburgh Postnatal Depression Scale (EPDS). The scale was developed by Cox to measure maternal depression [47]. The Chinese version of EPDS was adopted by Lee et al. [48]. This scale has 10 items in total, and each item adopts 4-point Likert scale which is counted as 0–3 points, so the total score range is 0 to 30. The total score greater than 9 is considered as having postpartum depression symptoms, with higher score indicating greater depression. In this study, Cronbach’s alpha was 0.91.

### We’ll App intervention

**Fig. 1:**
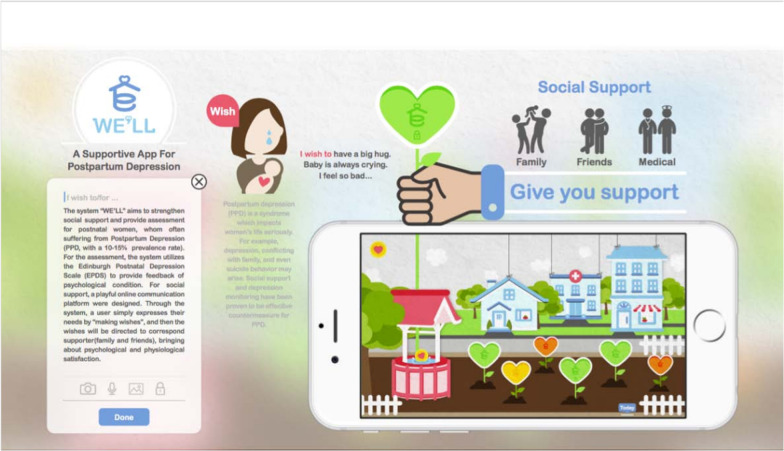
Interface of We’ll App

We’ll App (see Fig. 1) automatically collects compliance data, including the date, time, and name of each session that participants take part in, identified by the researcher. We’ll App transmitted these compliance data to the researchers every two weeks during the study. Using this data, researchers calculated the percentage of days that each participant used the app during the 8-week intervention [49].

The We’ll App aims to provide mindfulness and perceived social support interventions for puerperae during childbirth, with two main goals: (1) to provide guidance for puerperae on mindfulness meditation; (2) Provide perceived social support for puerperae.

The We’ll App has four components:(1) mindfulness, perceived social support, maternal self-efficacy, depression detection tools, and preventive health education; (2) postpartum women's social network: a social network composed of informal members of family, friends and puerperae, who can seek help or provide help to others; (3) wish garden area: wish flowers represent the expectations of puerperae, which family, friends and other puerperae can view and respond; (4) text introduction to the method of mindfulness meditation, which include: (a) body scanning: With eyes closed and gradually relaxing, participants directed their attention and observed the feelings, scanning one by one in a certain order (from head to foot or from foot to head) and becoming aware of the feelings of different parts of the body; (b) meditation, which involves observing a person's breath, feelings, emotions, sounds and thoughts; (c) mindful walking: it involves walking slowly and carefully, feeling the sensation of your feet on the ground as you walk, and being consciously aware of every detail as you walk [50, 51].

### Procedure and design

"We’ll App helps puerperae better understand themselves and solve their current problems." which was said in a recruitment advertisement posted at a maternity clinic in a Chinese hospital. Pregnant women about to give birth who were interested and eligible to participate in our study provided their registration information. Inclusion criteria: (1) voluntary participation in the study; (2) ages 20–40; (3) pregnant 36–38 weeks; (4) diagnosis of pregnancy; (5) no examination and treatment affecting endocrine function within half a year; (6) be able to use a smart phone for more than 3 years, can skillfully use various mobile phone applications. Exclusion criteria: (1) severe physical disease and mental disorder; (2) experience in mindfulness training; (3) are taking anti-anxiety or antidepressants. (4) High-risk pregnancy status was diagnosed by obstetrician. We then randomly assigned 130 pregnant women to either the App group (We’ll App intervention group) or the control group (Waiting group). To maintain participation and control attrition rates, there was a trial fee before and after the experiment, with each participant receiving 50 RMB at the start of the survey to boost their motivation. If they use the App for more than one hour a day during the 8-week App intervention period (the daily App use time of each user can be seen through the system background), they will receive 100 RMB test tokens at the end of the 8-week period, and those who quit during the 8-week period or not meet the daily App usage time will not receive the 100 RMB test tokens. Sample size was determined with G*Power. With 2 groups and 4 measurements, considering f = 0.3 at α = 0.05, 80% of power, and a medium effect size of correlation among repeated measures. The recommended sample size for ANOVA with repeated measures was 100, and we expected a 30% attrition rate from T0 (pre-test) to T1 (post-test), so we attempted to recruit at least 130 participants.

**Fig. 2:**
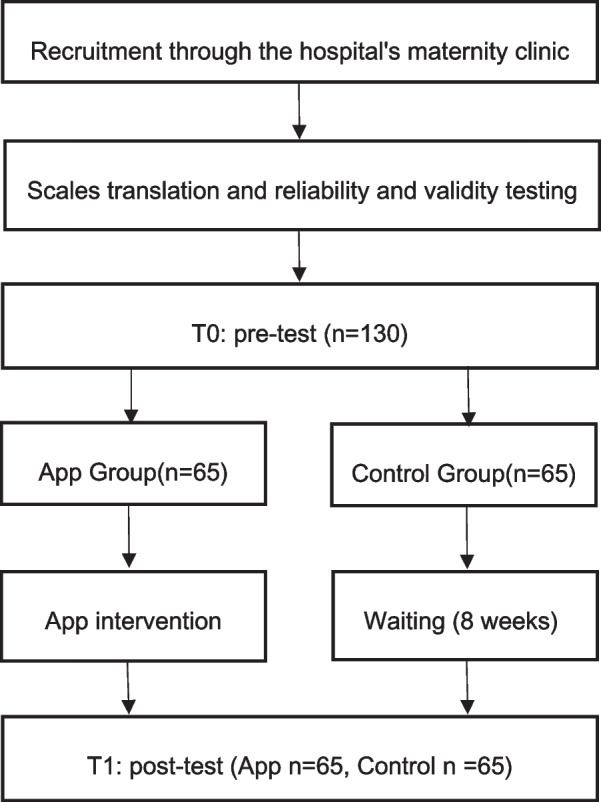
Procedure flow chart

The pre-test was conducted in the second week after delivery, and participants in the App group began to use the We’ll App immediately after the pre-test. Participants completed an online consent form via a secure online survey platform, provided demographic information and completed the following questionnaires (pretest): (1) the mindful awareness scale (MAAS); (2) self-efficacy Scale (PMPS); (3) Edinburgh Postpartum depressive symptoms Scale (EPDS); (4) perceived social support scale (MSPSS). It takes about 20 min to answer the questionnaire. App group received App intervention 3 times a week for a total of 8 weeks, while the control group did not receive any intervention. The experimental group was invited to participate in a 20-min webinar that provided (1) an overview of how to use mobile app, and (2) eight weeks of project components (such as meditation) and expectations (i.e., 24 days of daily meditation and responding to daily App posts). All participants began the 8-week study on the Monday following the webinar. Post-tests were performed immediately after the 8-week intervention, approximately 10 weeks postpartum. At the end of the intervention period (week 8), participants completed the same questionnaire again and received an additional 100 RMB compensation. The online funnel reporting procedure was followed in this study. Participants were asked if they knew the purpose of the study and the topic being investigated, and if they knew the same questions in the pretest and the posttest. In order to recruit subjects who were blind to the experimental conditions and naively practiced the App, funnel reporting helped to obtain homogeneous samples in both groups. Participants had the opportunity to anonymously record any number of questionnaires and can quit at any stage. The study was approved by the Ethics Committee of Chang gung University (IRB No: 202001014B0D001) and the protocol was carefully reviewed to ensure that it complied with the ethical guidelines of the psychological association in republic of China (The procedure flow chart is shown in Fig. 2).

### Data analysis

**Fig. 3:**
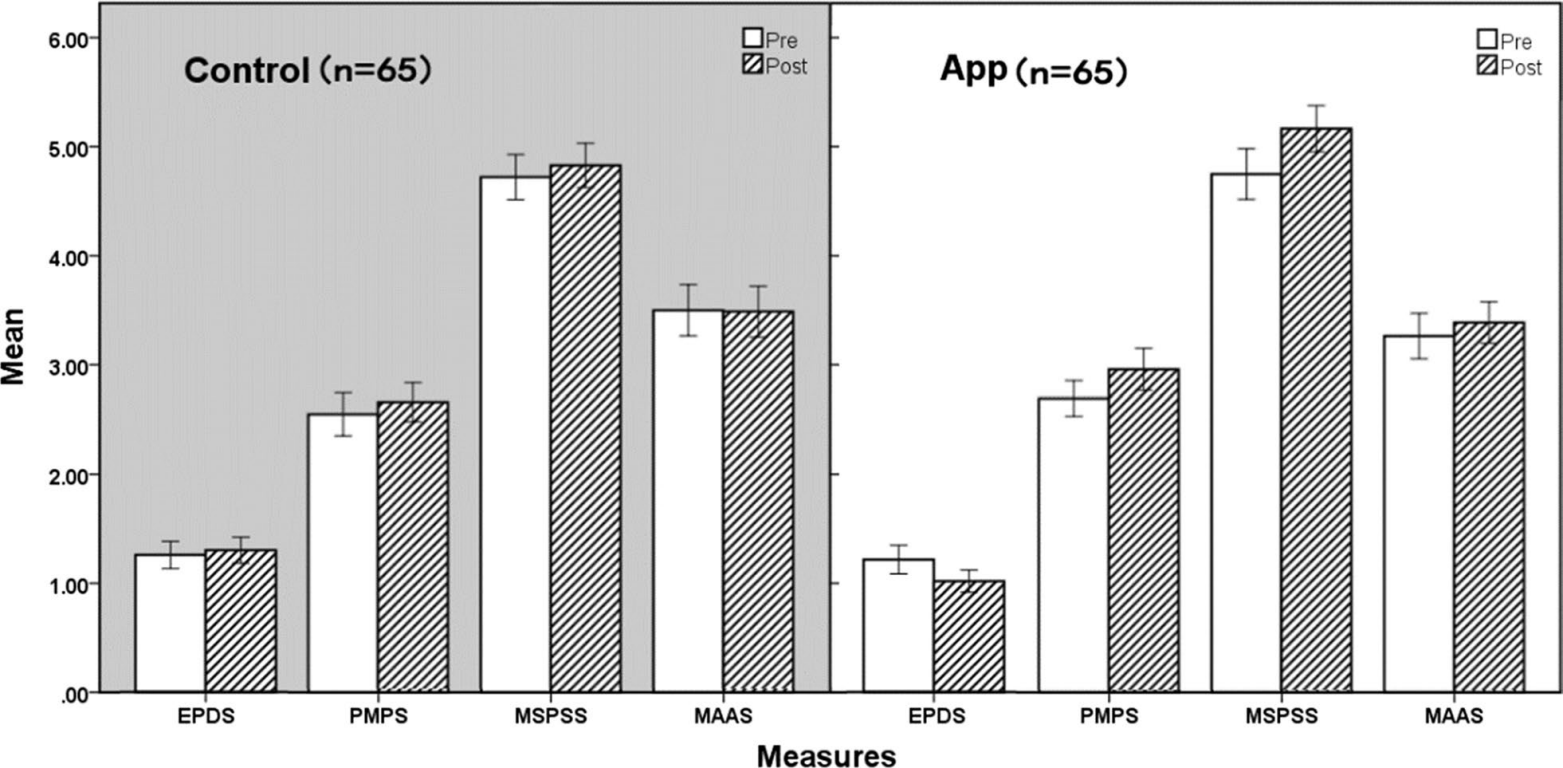
Comparison of 4 measures between App and Control Group. EPDS, Edinburgh Postnatal Depression Scale; PMPS, perceived maternal parental self-efficacy tool; MSPSS, perceived social support scale; MAAS, Mindfulness Attention Awareness Scale

IBM SPSS 23 software was used for statistical analysis. The confidence interval was set at 95%, and the significance level was set at 0.05. ANOVA with repeated measures was used to compare the differences pre and post intervention and between groups. Descriptive statistics were used to describe the distribution of basic data. In order to compare and illustrate each scale in the same figure (see Fig. 3), the average score of each item rather than the total score of each scale was used for statistical processing in this study (see Table 2). For example, EPDS used 4-point Likert score for evaluation, and the score range of each item is 0–3, so the average score of each item in EPDS was between 0–3.

**Table 2:** Means and standard deviations for each measure of each group, pre and post assessment

Group	Measures	Mean (SD)		
		T0	T1	T1–T0
APP (n = 65)	EPDS	1.308 (0.569)	1.094 (0.441)	− 0.214 (0.378)***
	PMPS	2.727 (0.669)	2.996 (0.781)	0.269 (0.360)***
	MSPSS	4.810 (0.949)	5.232 (0.866)	0.422 (0.712)***
	MAAS	3.305 (0.849)	3.430 (0.776)	0.126 (0.683)
Control (n = 65)	EPDS	1.352 (0.543)	1.399 (0.516)	0.047 (0.468)
	PMPS	2.552 (0.795)	2.662 (0.715)	0.111 (0.510)
	MSPSS	4.719 (0.837)	4.827 (0.818)	0.108 (0.567)
	MAAS	3.502 (0.948)	3.489 (0.940)	− 0.013 (0.794)

T0, pre-test; T1, post-test; EPDS, Edinburgh Postnatal Depression Scale; PMPS, perceived maternal parental self-efficacy tool; MSPSS, perceived social support scale; MAAS, Mindfulness Attention Awareness Scale

***p < 0.001

## Results

Four 2 (Group Type: APP, Control) × 2 (Time: T0, T1) ANOVA with repeated measures (Fig. 2) was conducted on the postpartum depressive symptoms (EPDS), parenting self-efficacy (PMPS), perceived social support (MSPSS), and mindfulness (MAAS). The descriptive statistics are presented in Table 2 and Fig. 3.

For postpartum depressive symptoms, there was a significant main effect of Time: F (1,128) = 5.005, *p* = 0.027, η2p = 0.038; and there was a significant main effect of Group Type: F (1,128) = 4.432, *p* = 0.037, η2*p* = 0.033; and there was a significant interaction between Time and Group Type: F (1,128) = 12.216, *p* < 0.001, η2*p* = 0.087. The results suggest that the APP intervention significantly reduced the subjects' levels of postpartum depressive symptoms, which is consistent with the hypotheses.

For parenting self-efficacy, there was a significant main effect of Time: F (1,128) = 24.094, *p* < 0.001, η2*p* = 0.158; and there was a significant main effect of Group Type: F (1,128) = 4.200, *p* = 0.042, η2*p* = 0.032; and there was a significant interaction between Time and Group Type: F (1,128) = 4.190, *p* = 0.043, η2*p* = 0.032. As can be seen from this result, the APP intervention significantly improved subjects' levels of parenting self-efficacy, which is consistent with the hypotheses.

For perceived social support, there was a significant main effect of Time: F (1,128) = 22.010, *p* < 0.001, η2*p* = 0.147; *p* = 0.407; and there was a significant interaction between Time and Group Type: F (1,128) = 7.749, *p* = 0.006, η2*p* = 0.057; but there was no significant main effect of Group Type: F (1,128) = 3.074, *p* = 0.082. These results revealed that the APP intervention can significantly help to improve subjects’ levels of perceived social support, also consistent with the above hypotheses.

For mindfulness there was no significant main effect of Time: F (1,128) = 0.755, *p* = 0.386; and there was no significant main effect of Group Type: F (1,128) = 0.829, *p* = 0.364; and there was also no significant interaction between Time and Group Type: F (1,128) = 1.137, *p* = 0.288. The results suggest that the APP intervention did not significantly improve the participants' mindfulness, which is not consistent with the hypotheses.

Our hypotheses stated that APP should reduce postpartum depressive symptoms; and increase parenting self-efficacy, perceived social support and mindfulness. As the results showed that H1, H2, and H3 were supported, but H4 was not supported.

The proportion of the total score of EPDS greater than 9 between the two groups is shown in Table 3.

**Table 3:** Comparison of total EPDS score greater than 9 between the two groups

Test	Total	App group	Control group	χ^2^	*p*
Pre	96 (73.8%)	47 (72.3%)	49 (75.4%)	0.451	0.502
Post	93 (71.5%)	41 (63.1%)	52 (80.0%)		

EPDS, Edinburgh Postnatal Depression Scale

Chi-square test results showed that We’ll App intervention for 8 weeks did not significantly reduce the number and proportion of subjects with postpartum depression symptoms, but significantly reduced the mean score of EPDS, and still had a significant effect on improving the postpartum depressive symptoms of subjects.

## Discussion

### App can reduce postpartum depressive symptoms

Perceived social support is a general term for the emotional or practical help that an individual feels or gives to himself. The results of this study indicate that good perceived social support can effectively reduce postpartum depressive symptoms. The decrease of perceived social support on postpartum depressive symptoms comes from the following three aspects: (1) Tangible support, substantive support plays an extremely important role in the process of the puerperae adapting to the maternal role. In this study, subjects often made wishes to their families, such as taking care of them, organizing the housework, directly helping them take care of the baby and providing them with direct material help, etc. Such support from the family is an effective way to reduce the life burden and stress of the puerperae and prevent and reduce postpartum depressive symptoms. And puerperae often regard their husbands' active involvement in baby care and household chores as a sign of "love" [52].The App provides women with information to help them learn how to care for their babies, how to care for themselves and how to juggle the roles of "mother" and "wife" in family life. Most of the puerperae in this study lived alone with their husbands after delivery and most of them had little experience in caring for their babies. The postnatal period is indeed a very difficult time for puerperae due to the lack of experience and knowledge of childcare and self-care and a time when there is an urgent need for guidance [53]. However, due to a shorter hospital stay after childbirth, it is difficult for nurses to provide a large amount of information in a short period of time, and postpartum home care and community care at present in China is not perfect. As a result, the difficulties faced by the postpartum period are not met by the substantial support women urgently need, resulting in mental stress and increasing the risk of experiencing postpartum depressive symptoms. The results of this study show that preventive health education can play a positive role in preventing and reducing postpartum depressive symptoms in primiparas [54]. (2) Ascription support, puerperae have clear expectations for attribution support after childbirth. The use of App provides them with more diverse sources for attribution support. Although it cannot completely replace real perceived social support, it can be an important supplement to real perceived social support. Attributional support is one of the most important types of perceived social support that mothers can obtain through the Internet [55]. In terms of attribution support, network perceived social support is considered to provide more comprehensive and diversified attribution because of the heterogeneity of virtual community members. Compared with traditional intergenerational support, maternal mothers are more inclined to obtain information from peers through network communication, which makes the intergenerational relationship in the family present a different appearance from the traditional elder-centered relationship, and is a form of "cultural feedback" under the background of new media [56]. Attributional support has a positive effect on postpartum depressive symptoms and strengthens women's identity of their own motherhood. The cross-temporal nature of the Internet makes it an important tool for women who cannot live with their families of origin or live apart from their friends because of social mobility. In addition, communication with peers in cyberspace can help women realize that they are not the only "new mothers" and that they are not the only ones facing problems, and this self-compassion can play a positive role in preventing and reducing postpartum depressive symptoms in puerperae [57]. (3) Emotional support refers to giving affirmation, recognition, praise and encouragement to the behavior of the object of support. It conveys a message that makes the puerperae feel capable of assuming a social role. Lack of emotional support can lead to underestimation or denial of self-worth [58]. In the early postpartum period, women are often vulnerable and sensitive to the attitudes and evaluations of others. Lack of emotional support easily leads to their negative emotions. On the other hand, if someone can recognize and encourage the efforts and progress of the women during this period and help them face the challenges of childbirth with confidence and make them feel valued, puerperae can avoid the symptoms of postpartum depressive symptoms [59].

### App can improve self-efficacy

The results of this study show that good perceived social support can effectively support the transition and development of postpartum women's mother role, and confidently face and overcome many postpartum difficulties and challenges. The improvement of perceived social support on self-efficacy comes from the following three aspects: (1) Tangible support: provide substantive support, such as materials, information or services. This form of perceived social support includes specific, direct ways for people to help puerperae. The puerperium has just finished the meticulous care of the family, and gradually began to take care of the baby independently. Due to the lack of direct parenting experience, the puerperae needs the help of parenting knowledge and skills provided by the App, so that they can successfully complete the baby care and other childcare work. Substantial support involves the promotion of postpartum rehabilitation, newborn care and disease prevention related information, and maternal physical and psychological changes, coupled with the lack of professional guidance during the separation of mother and child, make them more eager for their loved ones, especially professional care [60]. App provides them with professional parenting knowledge and skills, which improves their cognition of parenting knowledge, so their self-efficacy is improved [61]. (2) Ascription support: belongingness support is a type of support that gives the puerperae a sense of social belonging, also known as accompany support. This can be seen as the presence of peers participating in shared social activities. Puerperae need to share their lives, need to belong, need to accept others, need to identify with others. When this is satisfied, the woman usually feels satisfied, warm and safe [62]. Puerperae get self-efficacy by observing the behavior of others. The more similar the role models are, the stronger the self-efficacy of individuals will be. If other mothers successfully overcome postpartum emotional disorders, a demonstration effect will be formed, and the mothers in the same community will strengthen their self-belief and believe that they can also overcome postpartum emotional disorders [63]. (3) Emotional support: provide empathy, care, love, trust, acceptance, intimacy, encouragement or caring that lets women know they are valued. Practical emotional support can improve maternal self-efficacy and encourage them to cope with their new role in a new life. Therefore, appropriate emotional support can be transformed into self-motivation and self-efficacy of puerperae, so as to sustainably motivate them to face various pressures in postpartum life [64]. Community members' careful companionship, listening and communication unconsciously increase the satisfaction of them, and in the process of role change, community members' full help is also an important reason for puerperae to establish a high level of self-efficacy [65].

### App don't improve mindfulness

Many maternal feedback, as one who have never been exposed to mindfulness meditation, if they just read the mindfulness practice instructions text in the App, they will not be able to master the specific methods of mindfulness practice. Before carrying out daily mindfulness training, unified lectures should be offered to enable puerperae to have a preliminary understanding of mindfulness training, understand its origin, mechanism of action and forms of daily training, so as to facilitate the development of training. Improving maternal awareness of the scientific nature of mindfulness training, understanding the mechanism of mindfulness training, and enhancing the importance of mindfulness training are the important basis for ensuring the effect of mindfulness training [66].

At present, most of the mindfulness training instructions are carried out by the West, and the Eastern Buddhism has many years of mindfulness meditation practice. In terms of the explanation of mindfulness practice methods, the simple translation of western training instructions cannot completely conform to the thinking and language habits of puerperae. Therefore, when introducing mindfulness training into maternal mental health education, we should pay attention to the differences in order to make it easier for trainers to understand and accept [67]. At the same time, innovations should be made in training guidance language, training content and training methods, and training subjects with parenting characteristics should be added, so that the content and effects of mindfulness training can be better combined with the learning of parenting skills. Medium of mindfulness training should be further development of audio-visual animation guided version of mindfulness, which is convenient for puerperae to develop mindfulness training anytime and anywhere, guide them blend in mindfulness of the individual parts of life, mindfulness wash gargle, mindfulness to walk and so on, make it accord with the characteristics of low time cost, low cost money, so they can better improve maternal mindfulness [68, 69].

### App can improve perceived social support

In addition to early postpartum physical care, postpartum women also need to care for their newborn, which will reduce opportunities to go out and socialize. Through this app, puerperae can see the larger world, not just their own negative emotions, and this app can help reduce isolation and depression and improve perceived social support and self-esteem. Perceived social support through social connection and interpersonal exchange is seen as one of the most important benefits of online health activities. Perceived social support through social connection and interpersonal exchange is seen as one of the most important benefits of online health activities [49]. According to perceived social support theory, supportive interactions, together with perceived social support itself, can protect mothers from the health consequences of stress, increase adherence to treatment and promote recovery. We’ll App provides women with three types of perceived social support when they are under health stress: emotional support, Ascription support, and substantial support [70]. Emotional support refers to the degree to which the puerperae is respected, supported, understood and satisfied in society. Maternal mood is in an unstable state, they worried about their husband's infidelity, worried about their work, worried about the child after the inability to raise, and so on, which will make women in a state of depression, mood fluctuations. Over time, there will be a sudden release of this mood in the postpartum, leading to the occurrence of postpartum depressive symptoms. The use of App makes every bad emotion of puerpera, such as loneliness and helplessness, be responded to and released, thus enhancing perceived social support [71]. Ascription support refers to the internal connection between an individual and the group to which she belongs, and is the definition, identification and maintenance of a specific group and its subordinate relationship. In the app, puerperae can get support and establish or maintain friendships. Participants are not constrained by the time, geographic, and spatial constraints that face-to-face groups often have; Individuals can send and receive messages at any time of the day or night. In addition, online support groups can bring more diverse puerperae together, providing different experiences, perspectives and sources of information. By helping puerperae connect with other puerperae, they can better cope with their own experience of postpartum depressive symptoms and learn more about the voices of others [72]. Substantial support is the tangible help that the puerperae receives from the App. Many mothers are unable to adjust to the new tasks after the birth of their child, unable to fully balance the heavy workload with caring for the baby. Therefore, it is easy to cause postpartum depressive symptoms. App provides a variety of substantial support for puerperae related to pediatric diseases, child health care, childcare education and other aspects [73].

## Research limitations and future studies

The limitations of this study are as follows: (1) samples are taken from hospitals in first-tier cities in China, and the sample size is not large enough due to the limited number of obstetrics and gynecology patients, scientific research funds and other factors. (2) Since postpartum depressive symptoms belongs to a specific depressed population, the results of this study may not be applicable to other depressed populations, resulting in low general value of the research results. (3) The analysis of self-efficacy and postpartum depressive symptoms was conducted on the overall scale, without analyzing its sub-dimensions. (4) The subjects of this study are mostly urban women with relatively high education level. Therefore, the conclusion of this study may be limited by demographic, and future studies can consider more extensive sample collection. (5) Due to the limitation of research funds, only text mindfulness descriptions were used in the App development process. In the future, audio-visual animated versions of mindfulness practice instructions can be developed.

In future studies, we will expand the sample collection scope, increase rural hospital maternal subjects, the four factors of maternal mindfulness, perceived social support, self-efficacy and postpartum depressive symptoms will be studied in greater depth, further explore whether perceived social support App has the same impact on the rest of the group of samples, to test the universality of the results. Future research will further explore the mechanisms by which mindfulness and perceived social support affect self-efficacy and depression. Due to the complex relationship between mindfulness, perceived social support, self-efficacy and depression, we need to conduct in-depth research on each factor and integrate various variables to further explore the relationship between mindfulness, perceived social support, self-efficacy and depression. As a future perspective, longitudinal studies with repeated follow-up could be added to test the durability of APP efficacy over time.

## Conclusions

Our study showed that We’ll App caused a significant increase in maternal self-efficacy and perceived social support and a significant decrease in postpartum depressive symptoms. The results shed light on the psychological effects of We’ll App and raise the possibility of clinical application. The mechanisms underlying the effects of We’ll App on self-efficacy, perceived social support, postpartum depressive symptoms, and other psychological constructs need to be further elucidated.

## Data Availability

The datasets during the current study are not publicly available due to privacy restrictions but are available from the first author on reasonable request.
